# Genetic Loci Associated with Allergic Sensitization in Lithuanians

**DOI:** 10.1371/journal.pone.0134188

**Published:** 2015-07-27

**Authors:** Ingrida Šaulienė, Jūratė Greičiuvienė, Laura Šukienė, Neringa Juškevičiūtė, Christian Benner, Auksė Zinkevičienė, Samuli Ripatti, Kati Donner, Denis Kainov

**Affiliations:** 1 Deptartment of Environmental Research, Siauliai University, Siauliai, Lithuania; 2 Department of Immunology, State Research Institute Centre for Innovative Medicine, Vilnius, Lithuania; 3 Institute for Molecular Medicine Finland (FIMM), University of Helsinki, Helsinki, Finland; 4 Public Health, University of Helsinki, Helsinki, Finland; 5 Wellcome Trust Sanger Institute, Hinxton, United Kingdom; Cincinnati Children's Hospital Medical Center, University of Cincinnati College of Medicine, UNITED STATES

## Abstract

Allergic rhinitis (AR) is a common and complex disease. It is associated with environmental as well as genetic factors. Three recent genome-wide association studies (GWAS) reported altogether 47 single nucleotide polymorphisms (SNPs) associated with AR or allergic sensitization (AS) in Europeans and North Americans. Two follow up studies in Swedish and Chinese replicated 15 associations. In these studies individuals were selected based on the self-reported AR, or AR/AS diagnosed using blood IgE test or skin prick test (SPT), which were performed often without restriction to specific allergens. Here we performed third replication study in Lithuanians. We used SPT and carefully selected set of allergens prevalent in Lithuania, as well as Illumina Core Exome chip for SNP detection. We genotyped 270 SPT-positive individuals (137 *Betulaceae* -, 174 *Poaceae*-, 199 *Artemisia*-, 70 *Helianthus*-, 22 *Alternaria-*, 22 *Cladosporium-*, 140 mites-, 95 cat- and 97 dog dander-sensitive cases) and 162 SPT-negative controls. We found altogether 13 known SNPs associated with AS (*p* ≤0.05). Three SNPs were found in Lithuanians sensitive to several allergens, and 10 SNPs were found in Lithuanians sensitive to a certain allergen. For the first time, SNP rs7775228:C was associated with patient sensitivity to dog allergens (F_A=0,269, F_U=0.180, P=0.008). Thus, careful assessment of AS allowed us to detect known genetic variants associated with AS/AR in relatively small cohort of Lithuanians.

## Introduction

Allergic rhinitis (AR) is common and costly disease [1]. It is estimated that roughly 30% people have an active allergy at any given time and at least 75% people develop an allergic reaction once in a life. Prevalence of AR varies widely between different countries, due to cultural, environment and genetic factors [2–4]. Prevalence rates for AR in United States, Japan, France, Germany, Italy, Spain, and United Kingdom range from 14% to 31%, but diagnosis rates for the disease are only 30–40% [5]. The prevalence of AR in the industrialized world is increasing, particularly in urban areas [6,7]. This could be associated with globalization and improved methods for diagnostics of AR [8].

AR is an allergic inflammation of the nasal airways which occurs when an allergen, such as pollen, mould or mites, is inhaled by an individual with a sensitized immune system [9]. Chronic AR may cause acute or chronic sinusitis, otitis, sleep disturbance or apnoea, dental problems, palatal abnormalities, asthma [10].

AR is initiated when an inciting allergen interacts with inflammatory cells, which infiltrate the nasal lining. Inflammatory cells promote IgE production by plasma cells. IgE production, in turn, triggers the release of mediators from mast cells, such as histamine, leukotrienes and cytokines. The mediators trigger AR symptoms [11].

AR is diagnosed in points of care using skin prick tests (SPT for immediate hypersensitivity testing), radioallergosorbent (RAST), total serum IgE, or total blood eosinophil count tests. SPT remains the least expensive method of testing which effectively diagnoses the allergies in over 80% AR patients [12].

AR could be prevented by allergen avoidance or its symptoms can be reduced by allergen immunotherapy. There are several classes of drugs for treatment of AR: antihistamines and corticosteroids, as well as second line of drugs: decongestants, cromolyn, leukotriene receptor antagonists, or nasal irrigation [13]. But these prevention and treatment options are often ineffective because of the inherited component of AR [14].

The inherited component of AR is supported by three recent genome wide association studies (GWAS) on Europeans and North Americans, as well as by two replication studies on Swedish and Chinese with AR or allergic sensitization (AS) and controls (S1 Table)[15–19]. For example, Ramasamy and colleagues identified three genetic loci significantly associated with self-reported (SR) or laboratory-diagnosed AS to grass [19]. They also identified 12 loci with suggestive associations. Bonnelykke and co-authors analysed susceptibility loci associated with AS to different allergens and identified 10 associations with genome wide significance [16]. At the same, time Hinds and co-authors identified 16 loci associated with self-reported (SR) cat, dust-mite and pollen allergies [17]. Thus, original GWA studies identified statistical associations of AR/AS with minor alleles of 47 SNPs. Importantly, only few associations were reproduced in the replication studies [15, 16, 18]. Low reproducibility could be associated with partly comprehensive nature of the original and replication studies, and broad-range of diagnostic tests and allergens used for AS/AR assessments. Here we used carefully selected set of allergens prevalent in Lithuania, skin prick test, and Illumina Core Exome chip to identify known genetic associations in relatively small cohort of Lithuanians.

## Materials and Methods

Volunteers participating in this study gave their written informed consent. Volunteers were asked to fill in questionnaire forms which covered general personal information, allergy triggers and related indicators of quality of life. All documents filled were stored safely in a drawer equipped with a lock and in protected computers. Medical Faculty of Vilnius University review board and Vilnius Regional Biomedical Research Ethics Committee approved this project (GSSAR-03-069) including consent procedure and sample collections (Decision No. 158200-13-633-200 from 2013-06-11). All the ethical issues were handled in accordance with the national and EU regulations (Directive 95/46/EC). Other relevant ethical issues, such as good clinical practice and respect of the Lithuanian legislation and guidelines were applied and the study has been conducted according to the principles expressed in the Declaration of Helsinki. No dual use or misuse issues were concerned.

We recruited 432 random citizens of Lithuania (mean age = 39.1 years; 276 females). Patients did not receive allergen-specific immunotherapy, corticosteroids, or antihistamines before the study. We performed SPT using common inhalant allergens: *Alternaria*, *Cladosporium*, cats and dogs dander, and mites, as well as pollens that are prevalent in Lithuania: *Betulaceae* (*Alnus*, *Corylus*, *Betula*, *Carpinus)*, *Poaceae* (*Dactylis glomerata*, *Anthoxanthum odoratum*, *Lolium perenne*, *Poa trivialis*, *Phleum pratense*) mixes, *Artemisia vulgaris*, and *Helianthus annuus* [20]. Phenolated glycerol-saline was used as a negative control and histamine solution (10mg/ml)–as a positive control. The allergens and controls were purchased from Stallergens (France). The skin prick test with allergenic extracts was performed on the surface of the forearm and it was considered valid if the histamine wheal was at least 3 mm larger than the saline wheal, and a skin test response was considered positive if the net orthogonal wheal diameter was at least 3 mm larger than that elicited by the saline control. Healthy non-allergic controls were identified as subjects with negative SPT.

Whole blood samples were collected from study volunteers using Vacutainer 4ml CPT tubes (BD, Finland). Genomic DNA was isolated using DNeasy Blood and Tissue kit (Qiagen, UK). Quality of DNA was determined by electrophoresis in 1% agarose gel and DNA concentration was measured using spectrophotometry. One sample was discarded because of low QC rate (<95%).

Genotyping was performed using HumanCoreExome-24 v1.0 DNA Analysis Kit following procedure provided by Illumina (Illumina Inc., San Diego, CA, USA) in Institute for Molecular Medicine Finland FIMM Technology Centre, University of Helsinki. The kit was chosen because it covers 23 SNPs found in previous GWAS studies on AR/AS. All genotypes were called with GenomeStudio v.2011.1 software. Variants with low call rates, signal intensity, quality scores and heterozygote excess were discarded from further analysis based on the following QC parameters: call rates <97%, cluster separation <0.3, AB R Mean <0.3, AB T Mean <0.2 or >0.8, heterozygote excess <-0.3 or >0.2, AA or BB T Dev >0.04 and AB T Dev >0.05. Altogether we excluded 8241 out of 547644 variants, which represents 1.5% of analyzed variants. Identity-by-state clustering was performed using PLINK v1.07 toolset. 5 samples were discarded from further analysis because decent test indicated that these samples were replicates. Thus, the overall genotyping success rate was 96.2–99.8%. No variants were discarded based on minor allele frequencies. 13 variants associated with AR/AS were checked manually. Gene set enrichment analysis (GSEA) was performed on-line at www.broadinstitute.org/gsea/index.jsp.

## Results and Discussions

**Fig 1:**
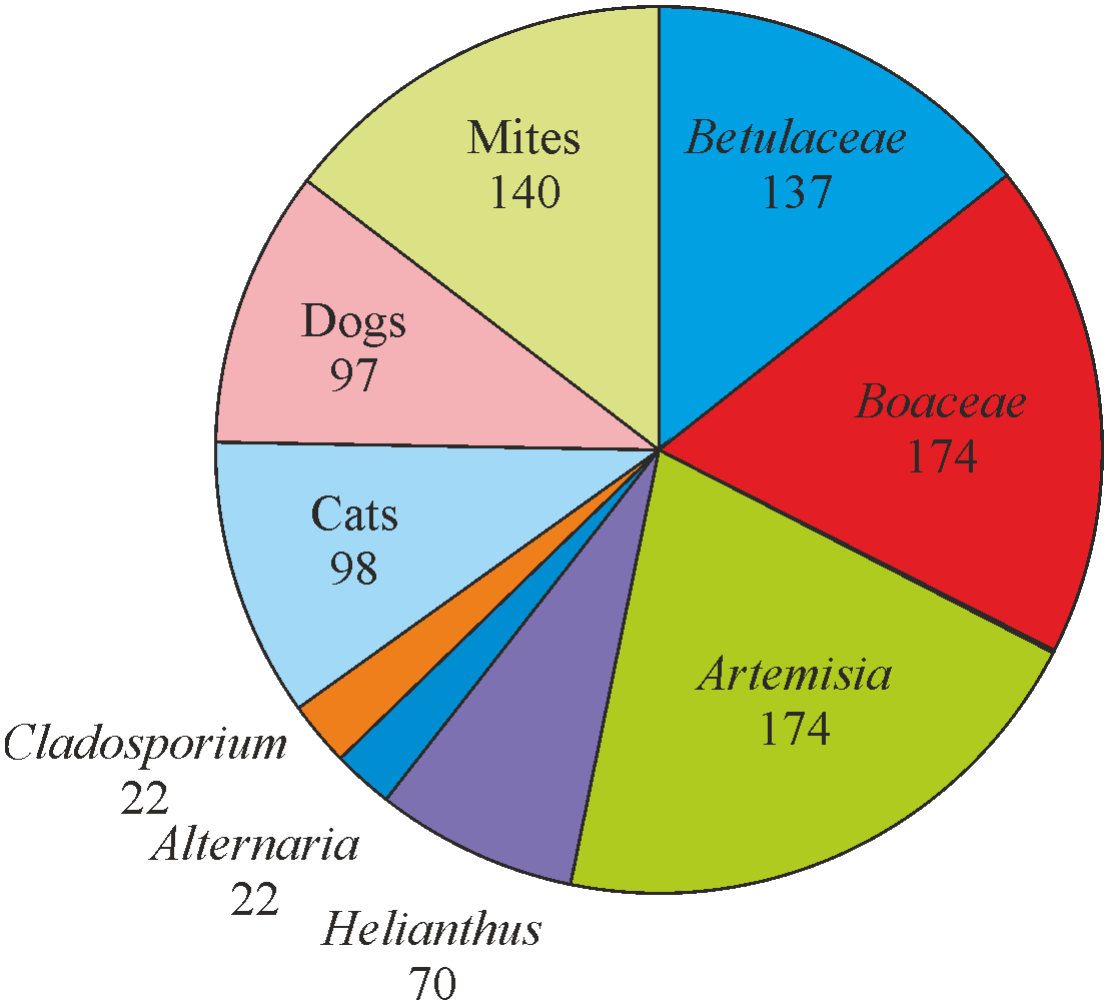
Pie diagram showing numbers of AS cases from Lithuanian study group sensitive to indicated allergens.

We analysed 432 individuals from Lithuania using SPT. We found 270 SPT positive and 162 SPT-negative individuals. According to questionnaires, among 270 SPT-positive patients, 121 reported AR, 58—asthma, and 49—both diseases, whereas among 162 SPT-negative individuals only one reported AR, and 8—asthma. Among SPT-positive individuals we found 137 *Betulaceae*-, 174 *Poaceae*-, 199 *Artemisia*-, 70 *Helianthus*-, 22 *Alternaria-*, 22- *Cladosporium*, 95 cat dander-, 97 dog dander-, 140 mite- sensitive cases (Fig 1).

**Fig 2:**
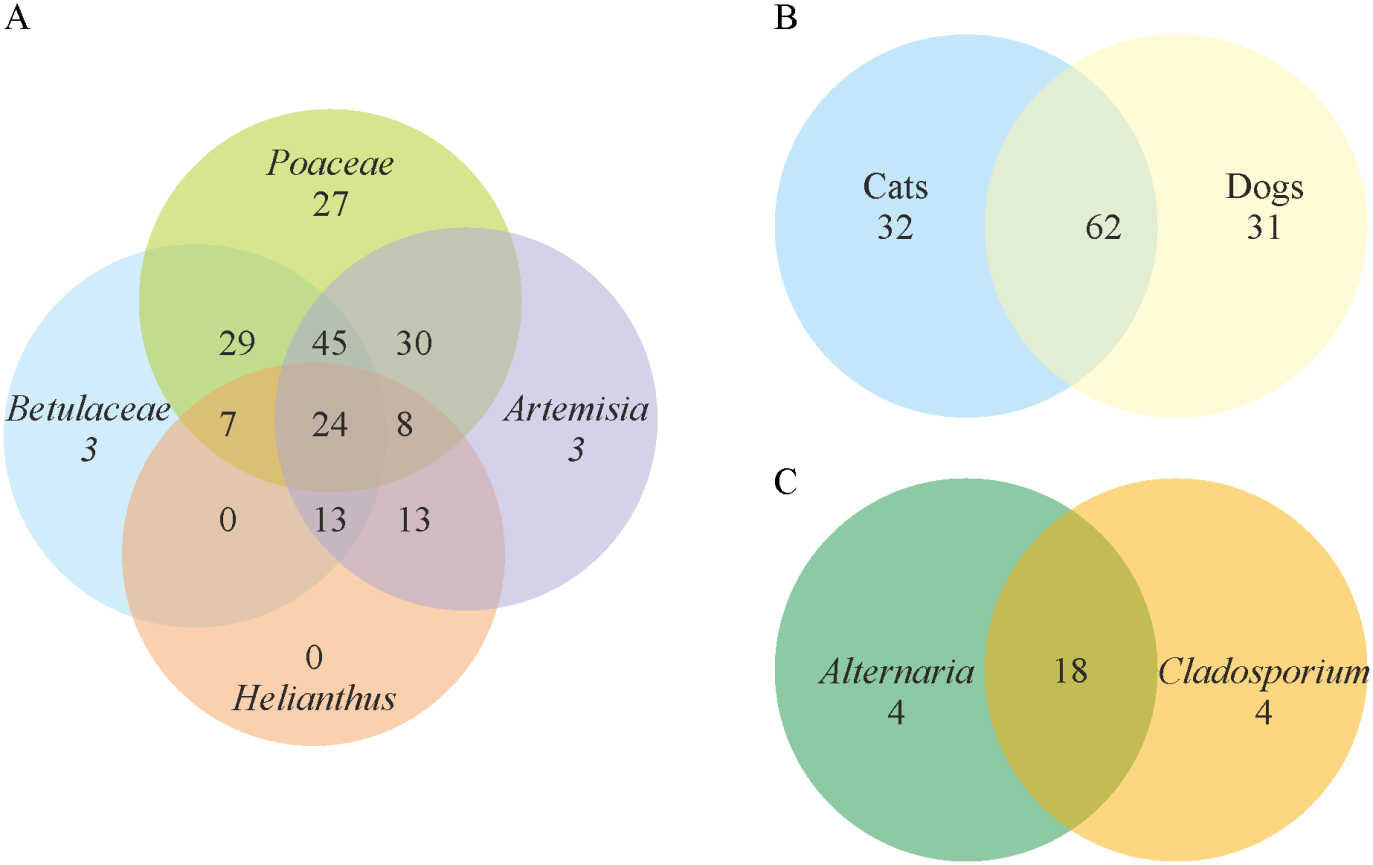
Venn diagrams showing the numbers of AS-positive patients from Lithuanian study group sensitive to one or more allergens of the same type. (A) AS to pollens. (B) AS to animal dander. (C) AS to moulds.

We found that only 24 individuals were sensitive to four pollen allergens, 18 individuals were sensitive to two moulds, and 62 individuals were sensitive to both cat and dog dander (Fig 2). Only 1 individual was sensitive to all 4 types of allergens. This result indicates that the etiology of AR is multifactorial and could be allergen-specific.

To find genetic associations with AS, we genotyped 432 individuals from our study group. We looked at known SNPs associated with AR in allergen-specific sub-cohorts. Out of 23 known SNPs covered by Human Core Exome chip we identified 13 SNPs (*p* ≤0.05) in our sub-cohorts (Table 1). In particular, we identified 3 SNPs shared between the individuals sensitive to different allergens. SNP rs10189629 (located within *IL18R1—[]–IL1RL2* gene *region)* was found in 5 different AS subclasses, SNP rs631208 (located within *GRIN2A —[]–C16orf72*) in 2 AS subclasses, and SNP rs7775228 (located within *HLA-DQA2—[]–HLA-DQB1* region) in 2 AS subclasses. We also identified 10 AS-specific SNPs. For the first time, SNP rs7775228 (*DQB1*) was associated with dog dander-specific AS, and SNP rs2069772 (*IL2*) was associated with sensitivity to *Alternaria*. Thirteen SNPs are located within gene regions which, according to GSEA, could be associated with NFkB-, Jak-STAT-, and IL12-mediated signalling pathways as well as antigen processing and presentation processes. Thus, we identified 13 known genetic variants associated with AS/AR in relatively small cohort of Lithuanians.

**Table 1:** Thirteen genetic variants associated with Lithuanian patient sensitivity to specific allergens (p≤0.05). SNP-SNP ID; CHR:BP—Chromosome: Physical position (base-pair); A1-Minor allele name (based on whole sample); F_A—Frequency of this allele in cases; F_U-Frequency of this allele in controls; A2—Major allele name; CHISQ—Basic allelic test chi-square; P—Asymptotic p-value for this test, OR—Estimated odds ratio (for A1, i.e. A2 is reference); L95-Lower bound of 95% confidence interval for odds ratio, U95-Upper bound of 95% confidence interval for odds ratio.

Allergen	SNP	Ref	CHR:BP	A1	F_A	F_U	A2	CHISQ	P	OR	SE	L95-U95
***Betulaceae***												
	rs10189629	[17]	2:102879464	A	0,118	0,173	C	4,091	0,043	0,641	0,2215	0,42–0,98
	rs1059513	[16]	12:57489709	C	0,088	0,052	T	3,904	0,048	1,762	0,2899	1,00–3,11
	rs887864	[19]	12:11158885	G	0,265	0,336	A	4,081	0,043	0,715	0,1665	0,52–0,99
***Poaceae***												
	rs6554809	[19]	5:13740976	T	0,079	0,144	C	8,147	0,004	0,507	0,2413	0,32–0,81
	rs6898653	[19]	5:115975656	G	0,167	0,226	A	4,258	0,039	0,687	0,1829	0,48–0,99
	rs631208	[19]	16:9399724	G	0,421	0,512	A	6,556	0,010	0,693	0,1434	0,52–0,92
***Artemisia***												
	rs10189629	[17]	2:102879464	A	0,126	0,181	C	4,647	0,031	0,654	0,1978	0,44–0,96
	rs1898671	[15,19]	5:110408002	T	0,332	0,260	C	5,026	0,025	1,411	0,1537	1,04–1,91
	rs7775228	[19]	6:32658079	C	0,167	0,229	T	4,870	0,027	0,675	0,1786	0,48–0,96
	rs631208	[19]	16:9399724	G	0,434	0,511	A	4,870	0,027	0,734	0,1406	0,56–0,97
***Helianthus***												
	rs7720838	[17]	5:40486896	G	0,341	0,461	T	6,704	0,010	0,605	0,1953	0,41–0,89
***Alternaria***												
	rs10189629	[17]	2:102879464	A	0,045	0,162	C	4,299	0,038	0,246	0,7303	0,06–1,03
	rs17388568	[17]	4:123329362	A	0,500	0,347	G	4,254	0,039	1,881	0,3108	1,02–3,46
	rs2069772	[19]	4:123373133	C	0,500	0,347	T	4,254	0,039	1,881	0,3108	1,02–3,46
***Cladosporium***												
	rs10189629	[17]	2:102879464	A	0,023	0,163	C	6,254	0,012	0,119	1,016	0,02–0,87
	rs4410871	[16]	8:128815029	T	0,091	0,237	C	5,004	0,025	0,323	0,5311	0,11–0,91
**Cats**												
	rs10189629	[17]	2:102879464	A	0,111	0,169	C	3,839	0,050	0,610	0,2545	0,37–1,00
**Dogs**												
	rs7775228	[19]	6:32658079	C	0,269	0,180	T	7,007	0,008	1,676	0,1963	1,14–2,46
**Mites**												
	rs10174949	[17,18]	2:8442248	A	0,193	0,259	G	4,427	0,035	0,682	0,1824	0,48–0,98

## Conclusions

GWAS is a widely used tool in search for genetic factors in allergic diseases. Yet it is not always straight forward to replicate original GWAS results. This could be seen from 3 original GWA and 2 replication studies, in which different allergens (inhaled, food, house dust-mite allergens), different detection tools (SPT, SR AR, IgE levels) and different demographics were used [15–19]. Here we performed third replication study in relatively small sub-cohorts of Lithuanians using a set of carefully selected allergens, prevalent in the region and SPT to assess the AS. We found altogether 13 known associations (*p* ≤0.05; S1 Table). In particular, we found 3SNPs in patients sensitive to multiple allergens, and 10 SNPs in patients sensitive to a certain allergen. Thus, careful assessment of AS allowed us to detect known genetic variants associated with AS/AR in relatively small cohort of Lithuanians.

Previous studies suggest that the SNP effects could be different for different antigens [15–19; 21]. We hypothesised that careful assessment of AS/AR could improve our ability to detect specific genetic variants. These warrant further replication, validation and functional studies on AR/AS focusing on allergen-specific AR/AS, which could deepen our understanding of the genetic risk factors, as well as lead to development of new strategies for more personalized prediction, prevention and treatment of AR/AS.

## Supporting Information

S1 Table Summary of supported evidence for allergy associated loci. Effect alleles associated with allergic disease in the three original and three replication studies are shown. In the original GWA studies, associations were identified in an IgE or skin prick tests of allergic sensitization (AS) without restriction to allergens (Bonnelykke et al. 2013), in a test of self-reported (SR) AS for cat, dust-mite, and pollen (Hinds et al., 2013), or in a test for SR AS or skin prick test or IgE test-diagnosed AS for grass (Ramasamy et al., 2011). In replication studies associations were identified in an IgE test for AS using different inhalant and food allergens (Nilsson et al., 2014), in a IgE test to house dust-mites (Andiappam et al., 2013), or in a skin prick test to pollen, cat, dog, and dust-mites (this study). Risk allele frequencies in cases (RAF), odds ratio (OR) for the risk alleles, and p-values are shown. NS indicates associations which were analysed but non-significant. If there is more than one association for genetic loci, than the most significant value is given.
(DOCX)
