# Supplementary material for: Genetic Loci Associated with Allergic Sensitization in Lithuanians
Source: PLoS One. 2015 Jul 27;10(7):e0134188. doi: 10.1371/journal.pone.0134188 (PMC4516305; doi:10.1371/journal.pone.0134188)
Supplement: S1 Table — Effect alleles associated with allergic disease in the three original and three replication studies are shown. In the original GWA studies, associations were identified in an IgE or skin prick tests of allergic sensitization (AS) without restriction to allergens (Bonnelykke et al. 2013), in a test of self-reported (SR) AS for cat, dust-mite, and pollen (Hinds et al., 2013), or in a test for SR AS or skin prick test or IgE test-diagnosed AS for grass (Ramasamy et al., 2011). In replication studies associations were identified in an IgE test for AS using different inhalant and food allergens (Nilsson et al., 2014), in a IgE test to house dust-mites (Andiappam et al., 2013), or in a skin prick test to pollen, cat, dog, and dust-mites (this study). Risk allele frequencies in cases (RAF), odds ratio (OR) for the risk alleles, and p-values are shown. NS indicates associations which were analysed but non-significant. If there is more than one association for genetic loci, than the most significant value is given. (DOCX) [file pone.0134188.s001.docx]

**Supporting Information**

**Genetic loci associated with allergic sensitization in Lithuanians**

### Ingrida Šaulienė^1^, Jūratė Greičiuvienė^1^, Laura Šukienė^1^, Neringa Juškevičiūtė^1^, Christian Benner^3^, Auksė Zinkevičienė^2^, Samuli Ripatti^3,4,5^, Kati Donner^3^, Denis E. Kainov^1,3^

**S1 Table. Summary of supported evidence for allergy associated loci.** Effect alleles associated with allergic disease in the three original and three replication studies are shown. In the original GWA studies, associations were identified in an IgE or skin prick tests of allergic sensitization (AS) without restriction to allergens (Bonnelykke et al. 2013), in a test of self-reported (SR) AS for cat, dust-mite, and pollen (Hinds et al., 2013), or in a test for SR AS or skin prick test or IgE test-diagnosed AS for grass (Ramasamy et al., 2011). In replication studies associations were identified in an IgE test for AS using different inhalant and food allergens (Nilsson et al., 2014), in a IgE test to house dust-mites (Andiappam et al., 2013), or in a skin prick test to pollen, cat, dog, and dust-mites (this study). Risk allele frequencies in cases (RAF), odds ratio (OR) for the risk alleles, and p-values are shown. NS indicates associations which were analysed but non-significant. If there is more than one association for genetic loci, than the most significant value is given.

| **Effect allele** | **Position** | **Gene region** | **Original studies** | | | | | | | | | **Replication studies** | | | | | | | | |
| --- | --- | --- | --- | --- | --- | --- | --- | --- | --- | --- | --- | --- | --- | --- | --- | --- | --- | --- | --- | --- |
|  |  |  | **Bonnelykke et al. 2013** | | | **Hinds et al. 2013** | | | **Ramasamy et al. 2011** | | | **Nilsson et al. 2014** | | | **Andiappan et al. 2013** | | | **This study** | | |
|  |  |  | **RAF** | **OR** | **P value** | **RAF** | **OR** | **P value** | **RAF** | **OR** | **P value** | **RAF** | **OR** | **P value** | **RAF** | **OR** | **P value** | **RAF** | **OR** | **P value** |
| **rs3771175:T** | 2:102960210 | *IL1RL-IL18R1* | 0,14 | 0,79 | 9,10E-06 |  |  |  |  |  |  |  |  |  |  |  |  |  |  |  |
| **rs9865818:A** | 3:188072513 | *LPP* | 0,59 | 0,88 | 3,40E-06 |  |  |  |  |  |  |  |  |  |  |  |  |  |  |  |
| **rs17616434:T** | 4:38812876 | *TLR1—TLR6– TLR10* | 0,78 | 1,24 | 3,80E-11 |  |  |  |  |  |  | 0,24 | 1,33 | 4,60E-03 |  |  |  |  |  |  |
| **rs17454584:A** | 4:123353432 | *IL2–ADAD1* | 0,74 | 0,88 | 9,50E-06 |  |  |  |  |  |  |  |  |  |  |  |  |  |  |  |
| **rs10056340:T** | 5:110190052 | *SLC25A46* | 0,83 | 0,82 | 3,20E-09 |  |  |  |  |  |  | 0,20 | 1,31 | 1,70E-02 |  |  |  |  |  |  |
| **rs6932730:T** | 6:31354182 | *HLA-B—MICA* | 0,82 | 1,18 | 7,90E-07 |  |  |  |  |  |  |  |  | ns |  |  |  |  |  |  |
| **rs6906021:T** | 6:32626311 | *HLA-DQB1* | 0,55 | 0,86 | 1,30E-08 | 0,48 | 1,1 | 7,10E-15 |  |  |  | 0,42 | 1,23 | 2,30E-02 |  |  |  |  |  |  |
| **rs4410871:T** | 8:128815029 | *MYC—PVT1* | 0,28 | 1,16 | 2,00E-08 |  |  |  |  |  |  |  |  | ns |  |  |  | 0,09 | 0,32 | 2,50E-02 |
| **rs1059513:T** | 12:57489709 | *STAT6* | 0,90 | 1,34 | 1,60E-10 |  |  |  |  |  |  |  |  |  |  |  |  | 0,09 | 1,76 | 4,80E-02 |
| **rs2056417:G** | 1:10581658 | *PEX14* |  |  |  | 0,69 | 1,07 | 3,70E-07 |  |  |  |  |  |  |  |  |  |  |  | ns |
| **rs10174949:G** | 2:8442248 | *ID2 –[]— RNF144A* |  |  |  | 0,72 | 1,07 | 1,00E-07 |  |  |  |  |  | ns |  |  |  | 0,19 | 0,68 | 3,50E-02 |
| **rs10189629:C** | 2:102879464 | *IL18R1 —[]– IL1RL2* |  |  |  | 0,86 | 1,16 | 1,80E-16 |  |  |  |  |  |  |  |  |  | 0,12 | 0,65 | 3,10E-02 |
| **rs10497813:G** | 2:198914072 | *PLCL1* |  |  |  | 0,48 | 1,08 | 6,10E-10 |  |  |  |  |  |  |  |  |  |  |  |  |
| **rs9860547:A** | 3:188128979 | *LPP* |  |  |  | 0,46 | 1,08 | 1,20E-09 |  |  |  |  |  | ns |  |  |  |  |  |  |
| **rs2101521:G** | 4:38811551 | *TLR6 —[]– TLR1* |  |  |  | 0,77 | 1,15 | 5,30E-21 |  |  |  | 0,22 | 1,33 | 5,50E-03 |  |  |  |  |  |  |
| **rs17388568:A** | 4:123329362 | *ADAD1* |  |  |  | 0,28 | 1,08 | 3,90E-08 |  |  |  |  |  |  |  |  |  | 0,5 | 1,88 | 3,90E-02 |
| **rs7720838:T** | 5:40486896 | *PTGER4 –[]— DAB2* |  |  |  | 0,58 | 1,08 | 8,20E-11 |  |  |  |  |  |  |  |  |  | 0,34 | 0,61 | 1,00E-02 |
| **rs1438673:C** | 5:110467499 | *CAMK4 —[]– WDR36* |  |  |  | 0,5 | 1,12 | 2,30E-20 |  |  |  |  |  |  |  |  |  |  |  |  |
| **rs9266772:C** | 6:31352113 | *MICA –[]— HLA-B* |  |  |  | 0,19 | 1,11 | 3,20E-12 |  |  |  |  |  |  |  |  |  |  |  |  |
| **rs6473223:T** | 8:81268155 | *ZBTB10 –[]— TPD52* |  |  |  | 0,36 | 1,07 | 7,70E-08 |  |  |  | 0,39 | 1,25 | 1,50E-02 |  |  |  |  |  |  |
| **rs7032572:G** | 9:6172380 | *IL33 –[]— RANBP6* |  |  |  | 0,17 | 1,12 | 1,70E-09 |  |  |  |  |  |  |  |  |  |  |  |  |
| **rs962993:C** | 10:9053132 | *CELF2 —[]– GATA3* |  |  |  | 0,58 | 1,07 | 1,50E-08 |  |  |  |  |  |  |  |  |  |  |  |  |
| **rs10893845:G** | 11:128186882 | *ETS1 –[]— KIRREL3* |  |  |  | 0,49 | 1,06 | 6,40E-07 |  |  |  |  |  | ns |  |  |  |  |  | ns |
| **rs1998359:G** | 14:38077148 | *SSTR1 —[]– MIPOL1* |  |  |  | 0,25 | 1,08 | 4,80E-08 |  |  |  | 0,3 | 1,26 | 1,90E-02 |  |  |  |  |  |  |
| **rs17228058:G** | 15:67450305 | *SMAD3* |  |  |  | 0,24 | 1,08 | 1,20E-08 |  |  |  |  |  |  |  |  |  |  |  |  |
| **rs7203459:T** | 16:11230703 | *CLEC16A* |  |  |  | 0,73 | 1,07 | 3,30E-07 |  |  |  |  |  |  |  |  |  |  |  | ns |
| **rs2107357:A** | 16:27410829 | *IL21R –[]— IL4R* |  |  |  | 0,14 | 1,09 | 3,30E-07 |  |  |  | 0,16 | 1,38 | 1,00E-02 |  |  |  |  |  |  |
| **rs9303280:C** | 17:38074031 | *GSDMB* |  |  |  | 0,52 | 1,07 | 8,90E-09 |  |  |  |  |  | ns |  |  |  |  |  |  |
| **rs6021270:T** | 20:50141264 | *NFATC2* |  |  |  | 0,94 | 1,16 | 6,90E-09 |  |  |  |  |  | ns |  |  |  |  |  |  |
| **rs6586513:C** | 1:17216331 | *CROCC –[]— MST1L* |  |  |  |  |  |  | 0,29 | 1,24 | 3,90E-06 |  |  | ns |  |  |  |  |  |  |
| **rs17513503:G** | 5:110146446 | *TSLP —[]– SLC25A46* |  |  |  |  |  |  | 0,01 | 1,39 | 1,20E-08 |  |  | ns |  |  | ns |  |  |  |
| **rs1898671:T** | 5:110408002 | *TSLP* |  |  |  |  |  |  | 0,38 | 1,15 | 5,20E-06 |  |  |  | 0,97 | 1,47 (AS) | 2,20E-02 | 0,33 | 1,41 | 2,50E-02 |
| **rs6898653:G** | 5:115975656 | *DTWD2 —[]– SEMA6A* |  |  |  |  |  |  | 0,22 | 1,23 | 1,00E-06 |  |  |  |  |  | ns | 0,17 | 0,69 | 3,90E-02 |
| **rs4724100:C** | 7:42264679 | *GLI3* |  |  |  |  |  |  | 0,45 | 1,14 | 2,20E-06 |  |  | ns |  |  | ns |  |  | ns |
| **rs2155219:T** | 11:76299194 | *LRRC32 —[]– C11orf30* | 0,47 | 1,2 | 1,80E-12 | 0,51 | 1,11 | 1,90E-19 | 0,47 | 1,22 | 1,20E-08 |  |  |  | 0,58 | 1,24 (AS) | 7,60E-04 |  |  | ns |
| **rs216518:A** | 14:60683430 | *PPM1A –[]— DHRS7* |  |  |  |  |  |  | 0,15 | 1,21 | 1,90E-06 |  |  |  |  |  | ns |  |  | ns |
| **rs887864:A** | 16:11158885 | *CLEC16A* |  |  |  |  |  |  | 0,63 | 1,16 | 1,10E-06 |  |  |  |  |  | ns | 0,27 | 0,72 | 4,30E-02 |
| **rs1044573:A** | 20:25206654 | *ENTPD6* |  |  |  |  |  |  | 0,51 | 1,15 | 9,70E-07 |  |  |  |  |  |  |  |  | ns |
| **rs6673480:T** | 1:51859242 | *EPS15* |  |  |  |  |  |  | 0,07 | 1,35 | 2,20E-06 |  |  |  |  |  | ns |  |  | ns |
| **rs1325195:A** | 1:179071756 | *ABL2* |  |  |  |  |  |  | 0,59 | 1,17 | 4,60E-06 |  |  |  |  |  | ns |  |  |  |
| **rs7617456:G** | 3:132727903 | *TMEM108 –[]— NPHP3* |  |  |  |  |  |  | 0,59 | 1,18 | 3,30E-06 |  |  | ns | 0,78 | 1,19 (AS) | 1,74E-02 |  |  | ns |
| **rs3860069:A** | 4:38862121 | *FAM114A1 –[]— TLR6* |  |  |  |  |  |  | 0,80 | 1,21 | 4,40E-05 | 0,19 | 1,45 | 5,60E-04 |  |  |  |  |  |  |
| **rs2069772:C** | 4:123373133 | *IL2* |  |  |  |  |  |  | 0,30 | 1,19 | 1,10E-06 |  |  |  |  |  | ns | 0,5 | 1,81 | 3,90E-02 |
| **rs6554809:C** | 5:13740976 | *DNAH5* |  |  |  |  |  |  | 0,84 | 1,29 | 3,30E-06 |  |  |  |  |  | ns | 0,08 | 0,51 | 4,00E-03 |
| **rs7775228:C** | 6:32658079 | *HLA-DQA2 —[]– HLA-DQB1* |  |  |  |  |  |  | 0,13 | 1,33 | 1,60E-09 |  |  |  |  |  |  | 0,27 | 1,68 | 8,00E-03 |
| **rs7789045:T** | 7:30494022 | *NOD1* |  |  |  |  |  |  | 0,55 | 1,15 | 6,20E-05 |  |  |  |  |  | ns |  |  |  |
| **rs631208:A** | 16:9399724 | *GRIN2A —[]– C16orf72* |  |  |  |  |  |  | 0,60 | 1,18 | 2,00E-06 |  |  |  |  |  |  | 0,42 | 0,69 | 1,00E-02 |
